# Multi‐institutional study on image quality for a novel CBCT solution on O‐ring linac

**DOI:** 10.1002/acm2.70023

**Published:** 2025-03-06

**Authors:** Luis Agulles‐Pedrós, R. Lee MacDonald, Amanda Jean Cherpak, Nayha Dixit, Lei Dong, Tianyu Zhao, Kundan Thind, Anthony Doemer, Boon‐Keng Teo, Shiqin Su, Alexander Moncion, James L. Robar

**Affiliations:** ^1^ Department of Medical Physics Nova Scotia Health Halifax Nova Scotia Canada; ^2^ Physics Department National University of Colombia Bogotá Colombia; ^3^ Physics Clinical Operations Varian Advanced Oncology Solutions Palo Alto California USA; ^4^ Department of Radiation Oncology University of Pennsylvania Philadelphia Pennsylvania USA; ^5^ Radiation Oncology University of South Florida Tampa Florida USA; ^6^ Radiation Oncology Henry Ford Health Detroit Michigan USA; ^7^ Radiation Physics Unviersity of Texas MD Anderson Cancer Center Houston Texas USA; ^8^ Department of Radiation Oncology University of Michigan Ann Arbor Michigan USA

**Keywords:** CBCT, image analysis, image quality, new technology, phantom image

## Abstract

**Introduction:**

This work presents a multi‐institutional study on image quality provided by a novel cone beam computed tomography (CBCT). The main goal is to investigate the consistency of imaging performance across multiple institutions.

**Methods:**

Phantoms for measuring relative electron density (RED) and image quality were sent to six institutions for imaging on Ethos and Halcyon units equipped with HyperSight CBCT. The imaging protocols included tube potential from 100 to 140 kVp and exposure from 80 to 800 mAs. Imaging performance was evaluated with regard to RED versus Hounsfield units (HU), uniformity, contrast‐to‐noise ratio (CNR), slice thickness, circular symmetry, modulation transfer function (MTF), and spatial resolution.

**Results:**

Among all institutions, some variability was observed among institutions in the RED‐to‐HU relationship, especially for RED values greater than 1, although no outliers were found (|*z*‐score| < 2 in all cases). In this range, RED/HU slopes were 475 ± 25 10^−6^ RED/HU at 100kVp, 505 ± 20 10^−6^ RED/HU at 125kVp, and 550 ± 20 10^−6^ RED/HU at 140kVp. Radial uniformity ranged from 1 to 7 HU, depending on protocol. Circular symmetry for two points 50 mm apart showed consistency within one‐pixel dimension. Integral nonuniformity was between 1 and 10, with no difference observed between vertical and horizontal dimensions. Contrast rods with 1% gave CNR = 0.5, 1 and 2 for 100(88), 125(176), and 140(528) in kVp(mAs), and contrast rods with 0.5% had CNR = 0.2, 0.4 and 0.8 for 100(88), 125(176), and 140(528) in kVp(mAs). Spatial resolution given by MTF at 10% and 50% yielded values of 0.55 ± 0.01 mm^−1^ and 0.35 ± 0.02 mm^−1^, respectively.

**Conclusions:**

This multi‐institutional analysis of CBCT imaging performance showed consistency in radial uniformity, circular symmetry, integral nonuniformity, contrast, and spatial resolution. Some variability was seen in the RED‐to‐HU relationship for RED > 1 depending on exposure. More data from different institutions would be necessary to establish more robust statistical metrics, which ensure quality parameters.

## 1 INTRODUCTION

Cone beam computed tomography (CBCT) is a critical external beam radiation therapy (RT) tool for precise image guidance. Techniques, such as intensity modulated RT (IMRT), volumetric modulated arc therapy (VMAT), and treatment approaches, such as adaptive RT (ART) particularly benefit from CBCT technology.^1^, ^2^, ^3^, ^4^ Despite its utility, historically, CBCT has exhibited inferior image quality compared to fan‐beam CT used in CT simulation for RT planning.^5^ This disparity is attributed to numerous factors including large solid angle for acceptance of scatter, which results in artifacts, nonuniformities, and inaccuracies in Hounsfield unit (HU) measurements.^6^, ^7^ Another factor to consider is the slower acquisition speed of CBCT, which leads to an increase in motion artifacts. In recent years, however, the CBCT image quality has been improved through techniques, such as iterative reconstruction, allowing for shorter acquisition times through shorter imaging arcs.^8^

Recently, the HyperSight CBCT imaging (Varian Medical Systems, Palo Alto, CA) system has been introduced and implemented on Halcyon and Ethos platforms, based in O‐ring linac, as well as conventional TrueBeam and Edge linac. This technology allows for larger detector size, faster acquisition times, and enhanced in‐room imaging.^9^ HyperSight is characterized by a CsI detector and advanced reconstruction software, enabling data acquisition within 6 s in Halcyon and Ethos platforms, substantially faster than standard CBCT.^9^ However, in addition to speed, image quality is critical, especially in the context of ART where CBCT must allow both accurate structure contouring and dose calculation. Performance criteria for CBCT systems in RT include image quality, HU accuracy, and acquisition efficiency. Preliminary investigations and technical specifications indicate superior image quality and HU accuracy compared to preceding CBCT systems, facilitating direct dose calculation.^10^, ^11^, ^12^ HyperSight has been evaluated under the ACR criteria and shows better performance than standard CBCT systems.^13^ Compared to the previous generation of CBCT imaging from the same manufacturer, HyperSight shows improved image quality.^14^ Given the novelty of HyperSight technology, there is a pressing need to evaluate its imaging performance deeper and broaden the scope of the results.^15^, ^16^

To date, no study has evaluated the variability of imaging performance of HyperSight among institutions. In this work, six institutions, including three Ethos and three Halcyon systems, were provided with the same image quality and an electronic density phantom for imaging with prescribed clinical protocols. This work presents an analysis of multi‐institutional image quality parameters, such as image uniformity, contrast, contrast‐to‐noise ratio (CNR), and HU versus relative electron density (RED) curves.

## 2 MATERIALS AND METHODS

### 2.1 HyperSight

HyperSight is a newly designed CBCT imaging detector integrated into Varian Halcyon, Ethos, TrueBeam, and Edge systems, with a detector of dimensions 86 × 43 cm^2^ (compared to the previous generation 43 × 43 cm^2^), oriented with the short axis along the cranial‐caudal direction.^17^ Its design incorporates a focused grid to minimize scatter and low‐noise electronics, enabling readout of up to 70 frames per second during continuous Kilovolt beam exposure. Operating in full‐fan mode due to its larger size, the source/detector pair accelerates through a 54° arc at a speed of 6 RPM in Halcyon and Ethos platforms. Acquisition can be completed in 5.9 s, starting 20° from posterior, over a rotation angle of 211°. Available CBCT reconstruction algorithms include Feldkamp Davis Kress (FDK) backprojection, iterative CBCT (iCBCT), iCBCT supplemented with Acuros CTS for scatter correction (iCBCT Acuros), and a metal artifact reduction (MAR) mode based on the iCBCT Acuros approach. The default reconstructed diameter is set at 53.8 cm. For consistency, this study exclusively utilized the iCBCT Acuros reconstruction algorithm.^6^, ^18^, ^19^

#### 2.1.1 Institutions

The study involved six institutions with the HyperSight system on the Ethos and Halcyon platforms. Nova Scotia Health Authority (NSH) in Halifax, Canada, Henry Ford Cancer Institute (HFord) in Detroit, USA, and The University of Michigan (UMich) in Michigan, USA, used the Ethos platform. The University of Pennsylvania (UPenn) in Philadelphia, USA, Washington University (WashU) in Saint Louis, USA, and Northeastern Oklahoma Cancer Institute (NOCI) in Oklahoma, USA, utilized the Halcyon platform.

### 2.2 Phantoms

**FIGURE 1:**
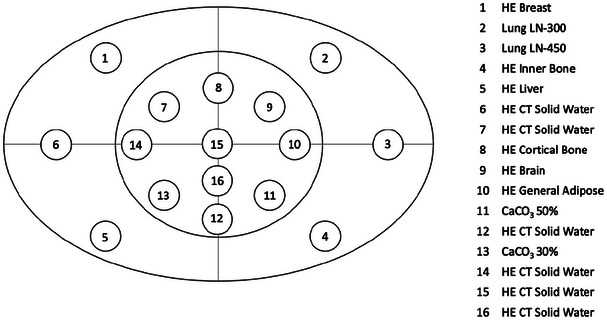
Rods distribution in the Sun Nuclear advanced electron density Phantom.^20^ ^.^

Two phantoms were employed for the study, one for measuring RED and another for measuring the image quality. The assessment of RED versus HU was conducted using the advanced electron density (AED) Phantom (Sun Nuclear, Melbourne, FL).^20^ This phantom includes 16 rods immersed in a cylinder‐like solid water frame with an elliptical cross‐section, measuring 40 cm wide and 30 cm high. It is an extended version recommended for broad beams covering 26.5 cm along the axial direction, since scatter can affect the measurements. The rods have dimensions of 3 cm in diameter and 16.5 cm in length, positioned along the axial direction. RED values for this study were: 0.28 (Lung LN‐300), 0.44 (Lung LN‐450), 0.94 (HE General Adipose), 0.97 (HE Breast), 1.00 (HE CT Solid Water), 1.02 (HE Brain), 1.05 (HE Liver), 1.16 (HE Inner Bone), 1.27 (CO_3_Ca 30%), 1.46 (CO_3_Ca 50%), and 1.78 (Cortical Bone). These values can be found in the manufacturer's manual.^20^ See Figure 1 for the location of each rod. Additionally, six solid water rods with a RED of 1.00 were placed in the center and periphery to mitigate nonuniformity artifacts; their average was considered for HU measurement.

Image quality metrics were assessed with the CatPhan 604 phantom (Phantom Laboratory, Salem, NY), which comprises a cylinder with four axial plane sections designed for analyzing various image quality parameters.^21^ Parameters, region of interest (ROI) size, and positioning adhered to standardized guidelines.^22^, ^23^

Section 1 consists of a uniform material with an approximate HU value of 10. This section is used to compare the effects of attenuation between the border and center parts of the phantom.

Section 2 comprises three sets of contrast rod targets measuring 40 mm in length with varying diameters ranging from 2 to 15 mm. Each set has a nominal contrast of 1%, 0.5%, and 0.3%, which gives around 10, 5, and 3 HU, respectively. To maintain consistency across protocols and institutions, only the 15 mm diameter rod was used for contrast calculation.

Section 3 provides four parameters for analysis. (i) Slice thickness is assessed by projecting two wires tilted 23° in the sagittal and two in the coronal planes. (ii) Circular symmetry is measured using the distances of four 3 mm diameter holes located at the corners of a 50 mm square. (iii) CT numbers are acquired from a set of 10 sensitometry targets composed of Teflon, Bone 50%, Delrin, Bone 20%, acrylic, Polystyrene, low‐density polyethylene (LDPE), polymethylpentene (PMP), Lung foam #7112, and two air targets. The sensitometry targets have a 15 mm radius. (iv) Finally, the modulation transfer function (MTF) is obtained by the Fourier transform of two axial projections of a 50 µm diameter wire along the axial direction (tilted 5°).

Section 4 has 15 sets of line pairs per cm (lp/cm) for spatial resolution test. The sets are made of 2 mm thick aluminum sheets and cast into the urethane background. In this work, the observable values of line pairs per centimeter and size gap were: 1, 2, 3, 4, and 5 lp/cm, and gap sizes 0.5, 0.25, 0.167, 0.125, and 0.1 cm, respectively.

Each section was identified using axial bead references included in the phantom. For analysis, five adjacent slices around each bead were visually inspected to ensure complete coverage of the calculated metrics, except for spatial resolution measurements. Before analysis, a meticulous visual examination ensured accurate bead localization and precise ROI delineation.

### 2.3 Imaging protocols

Imaging protocols are the Ethos and Halcyon defaults by anatomical site and are listed in Table 1. CBCT for planning (CBCTp) protocols provide a higher signal to noise ratio and are only available in Halcyon systems. This feature was introduced on Halcyon systems before broader implementation, such as in Ethos, which focuses on ART workflows reliant on AI‐based real‐time adaptation rather than fixed CBCT planning protocols.^1^, ^24^ Since only three institutions could provide CBCTp protocol data, this work did not analyze these. The results of three protocols are included in this work: 2. head, 3. thorax and 5. pelvis large. Since these protocols can be found in Halcyon and Ethos systems at the six institutions, they offer more robust statistical significance. Additionally, they were chosen to cover ranges of kilovoltage peak and milliampere‐seconds settings commonly used in practice. Due to its novelty, the results of the remaining protocols, including the CBCTp ones, can be accessed through the link provided in Appendix A.

**TABLE 1:** Acquisition protocol parameters.

Protocol	kV	mAs	Slice thickness
1. Breast	125	29	2 mm
2. Head	100	88	2 mm
3. Thorax	125	176	2 mm
4. Pelvis	125	469	2 mm
5. Pelvis large	140	528	2 mm
6. CBCTp head*	125	804	3 mm
7. CBCTp pelvis*	125	528	3 mm
8. CBCTp abdomen Lg*	140	880	3 mm

*Note*: The (*) denotes protocols exclusive to Halcyon systems. Protocols 2, 3, and 5 are referenced in this work; for complete results, please refer to the link provided in Appendix A.

Abbreviation: CBCTp, cone beam computed tomography for planning.

In the case of RED versus HU calibration images, the field of view (FOV) was set as: 538 mm (512 pixels) width, 538 mm (512 pixels) height, and 206 mm depth. CatPhan images were acquired with the following specifications: 538 mm (512 pixels) width, 538 mm (512 pixels) height, and 154 mm depth. Across all protocols, the iCBCT Acuros option was selected for reconstruction. Image analysis was conducted using MatLab.^25^

### 2.4 Image analysis

#### 2.4.1 RED versus HU

Following visual identification of each rod in the AED phantom, a circular ROI with a 10 mm radius was defined at each rod's position. Data within these ROIs were then averaged across five consecutive slices centered at the midpoint of the phantom axial dimension. The standard deviation of these values was then computed to estimate the HU error.

#### 2.4.2 Uniformity

Radial uniformity (RU), uniformity index (UI), and integral nonuniformity (InU) were evaluated within section 1 of the Catphan phantom. RU was determined by subtracting the mean HU value of the top, bottom, left, and right (ROI_borders_) from the HU at the center (ROI_center_) as follows:

$$\mathrm{RU}\;=\;\mathrm{mean}\left(\mathrm{RO}I_{\mathrm{borders}}\right)-\mathrm{RO}I_{\mathrm{center}}.$$

The uncertainty of RU was computed using the standard deviation across five slices centered in this section. Similarly to RU, UI was obtained by subtraction of the center, and borders normalized as a percentage with respect to the center ROI following

$$\mathrm{UI}=100\frac{\mathrm{mean}\left(\mathrm{RO}I_{\mathrm{borders}}\right)-\mathrm{RO}I_{\mathrm{center}}}{\mathrm{RO}I_{\mathrm{center}}}.$$

Detailed results are available in the Appendix A link. The error for UI was assessed analogously to RU. All ROIs were circular with a radius of 10 mm. Border ROIs were centered 20 mm from the phantom edge. For InU calculation, a centered horizontal and vertical line of section 1 of the Catphan phantom was considered. InU was derived from:

$$\mathrm{InU}=\frac{\mathrm{Max}-\mathrm{Min}}{\mathrm{Max}+\mathrm{Min}}$$

where Max and Min refer to the maximum and minimum values in vertical and horizontal projections, respectively. Error propagation evaluated the uncertainty per slice, and then the standard deviation across was computed from the five slices centered on section 1.

#### 2.4.3 Low contrast

Contrast was assessed in section 2 by calculation of CNR as follows:

$$\mathrm{CNR}=\frac{S_{\mathrm{ROI}}-S_{\mathrm{BKG}}}{\frac{1}{2}\left(\sigma_{\mathrm{ROI}}+\sigma_{\mathrm{BKG}}\right)}$$

where *S*_ROI_ is the signal in HU from the contrast rod ROI, *S*_BKG_ from the background, and *σ*_BKG_ and *σ*_ROI_ indicate the standard deviation within the background and contrast rod ROI, respectively. Background measurements were obtained from two ROIs of similar size located 20 mm above and below the center of the 15 mm rod in the radial direction to mitigate uniformity effects. All ROIs were circular with a radius of 10 mm to ensure homogeneous coverage of the target. Error estimation was determined from the standard deviation across five slices centered at section 2.

#### 2.4.4 Slice thickness

Slice thickness was obtained from the mean of the full width at half maximum (FWHM) of the four profiles of the 23° tilted wires of phantom section 3 (i). Uncertainty was calculated from the standard deviation of the four measurements.

#### 2.4.5 Circular symmetry

Circular symmetry was measured from the average of the four distances of the 50 mm square of section 3 (ii). The uncertainty was measured from the standard deviation of the average across five slices centered on section 3.

#### 2.4.6 CT numbers

CT numbers were acquired from 5 mm radius ROI in each sensitometry target of section 3 (iii), with error estimation derived from the standard deviation across five slices centered at section 3.

#### 2.4.7 Modulation transfer function

The MTF is obtained from the two axial projections of the wire of section 3 (iv) as follows: a 10 mm square ROI was defined centered at the wire. After recentering and averaging the two lateral projections of the square, the MTF is given by the Fourier transform of the resulting profile.^26^, ^27^, ^28^ Then, a linear interpolation of the values at 10% and 50% of the initial value was obtained. Uncertainty was computed from the differences in points used for the linear interpolation.

#### 2.4.8 Line pair per cm

A line was defined across each set of line pairs in section 4 to measure the spatial resolution in terms of line pairs per centimeter. A profile of each set was then fitted to obtain peaks, valleys, and widths. The MatLab (MathWorks, Natick, MA) peak and valley fitting algorithm called findpeaks was chosen to avoid visual subjectivity.^25^ The differences between peak and valley were ensured to be larger than 30% of the maximum value, although a visual inspection ensured a reasonable fitting.

## 3 RESULTS AND DISCUSSION

### 3.1 Relative electron density versus Hounsfield units

**FIGURE 2:**
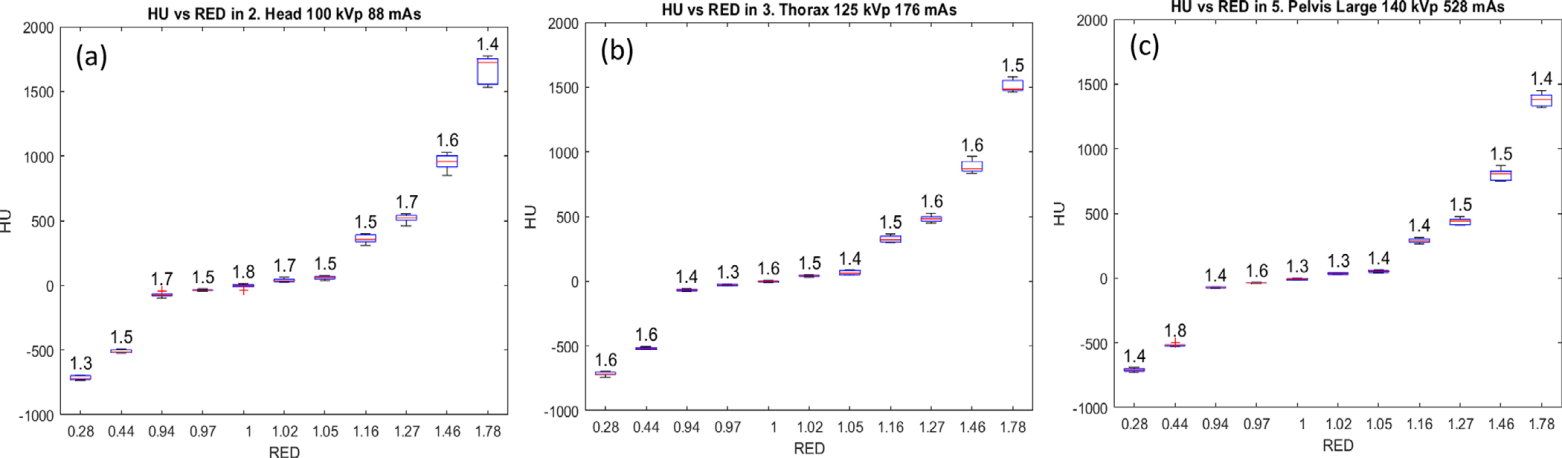
Multi‐institutional HU for different RED values using three reference protocols; (a) head, (b) thorax and (c) pelvis large. A *z*‐score test was conducted to identify outliers, and the maximum *z*‐score for each dataset is displayed above the respective box plot. HU, Hounsfield unit; RED, relative electron density.

HU versus RED data are shown for the three reference protocols in Figure 2, including data for all institutions. Although RED versus HU curves are vital for planning purposes, not all protocols are recommended for this end, and for Ethos and Halcyon platforms, HU accuracy is possible for 125 and 140 kVp, that is, used for the thorax and pelvis large protocols. The head protocol is also included here for comparison. The data show more consistent results among institutions at low RED values. For example, the standard deviation for RED = 0.28 is 16 HU, and 19 HU for RED = 0.94, both with the 100 kVp protocol, see Table 2. The other two protocols show even lower standard deviations. However, variations become noticeable at higher RED values within the same protocol due to slight differences in beam spectra across equipment. In the case of RED = 1.27, the standard deviation was around 30 in the three 100, 125, and 140 kVp protocols, respectively. The values of RED = 1.46 were 60, 50, and 50 HU for the 100, 125, and 140 kVp protocols, respectively. The largest RED (RED = 1.78) had standard deviations of 110, 50, and 50 HU for the 100, 125, and 140 kVp protocols, respectively. In any case, a *z*‑score test was conducted to identify statistical outliers. The maximum of the absolute value of the z‑score for each dataset is displayed above the respective box plot in Figure 2. All values were below 2 in all eight protocols listed in Table 1.

**TABLE 2:** Median and standard deviation of HU obtained across the six institutions from the eleven inserts with varying RED.

RED	0.28	0.44	0.94	0.97	1.00	1.02	1.05	1.16	1.27	1.46	1.78
Head	−721 ± 16	−515 ± 11	−75 ± 19	−35 ± 6	−3 ± 16	35 ± 14	61 ± 14	360 ± 30	520 ± 30	960 ± 60	1720 ± 110
Thorax	−720 ± 16	−518 ± 8	−68 ± 8	−29 ± 5	0.1 ± 6.3	40 ± 6	61 ± 16	320 ± 30	480 ± 30	870 ± 50	1480 ± 50
Pelvis L	−712 ± 14	−520 ± 10	−73 ± 5	−38 ± 3	−7 ± 6	33 ± 6	51 ± 9	290 ± 18	440 ± 30	800 ± 50	1380 ± 50

Abbreviations: HU, Hounsfield units; RED, relative electron density.

Regarding the absolute HU numbers, a decrease in HU was observed for large RED as tube potential increases, as shown in Figure 2. In the case of RED = 1.27, it was 522, 482, and 442 HU for the 100, 125, and 140 kVp protocols, respectively. In the case of RED = 1.46, the values were 957, 867, and 808 HU for the 100, 125, and 140 kVp protocols, respectively. Finally, for RED = 1.78, it was 1721, 1484, and 1312 HU for the 100, 125, and 140 kVp protocols, respectively. Similar behaviors have been reported with HyperSight.^12^, ^14^ Compared to a recent HyperSight work with the AED phantom, our results also agree with the data provided by Bogowicz et al.^15^ Although the bone inserts were positioned far enough apart to minimize the beam hardening effect, it was visually observed in some protocols, particularly at 100 kV. However, this effect did not extend to the closer inserts. A comparison of standard deviations revealed differences of up to 10% in the ROI's standard deviation. Still, this value was sometimes higher and at other times lower, so no clear effect of beam hardening could be established.

**FIGURE 3:**
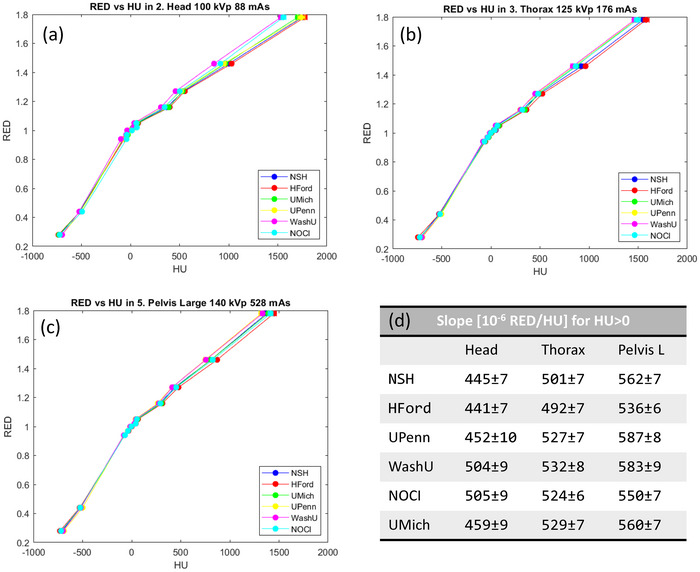
Multi‐institutional RED versus HU comparison. (a), (b), and (c) show the HU values at the head, thorax, and pelvis large protocols, respectively. (d) Slope fitting for RED≥1 across different protocols. Error bars are plotted in (a), (b), and (c); however, as errors are generally below 5%, these bars are not visible. HU, Hounsfield unit; RED, relative electron density.

Figure 3 shows the RED versus HU curves for the three reference protocols for all institutions. Piecewise linear fitting was performed for RED below and above 1 to determine slopes. In the Head protocol (see Figure 3a), two groups are observed among institutions (WashU and NOCI vs. others), clearly observable at HU over 500. This pattern is not reproduced in other protocols. This difference may arise because of the HU current dependence at low milliampere‐seconds. It has been noted that there is a pronounced HU dependence for milliampere‐seconds values below 150–200, depending on the kilovoltage peak (kVp) used. In the case of 100 kVp, a plateau is reached when more than 200 mAs is used. However, for 125 and 140 kVp cases, around 150 mAs are sufficient to reach the plateau.^29^

Figure 3d reports the slope values of the RED versus HU curve at RED > 1. While there are variations in slope within each protocol, slope values increase with higher energy, indicating reduced sensitivity to RED. Similar behavior have been observed in the data annexed by Bogowizc et al^15^: 530×10^−6^ and 554×10^−6^ RED/HU for 125 and 140 kVp, respectively. The intercept consistently remains at 1 in all cases, with a margin of error of approximately 1%. Refer to the link in Appendix A for further details.

### 3.2 Image quality

#### 3.2.1 Uniformity

**FIGURE 4:**
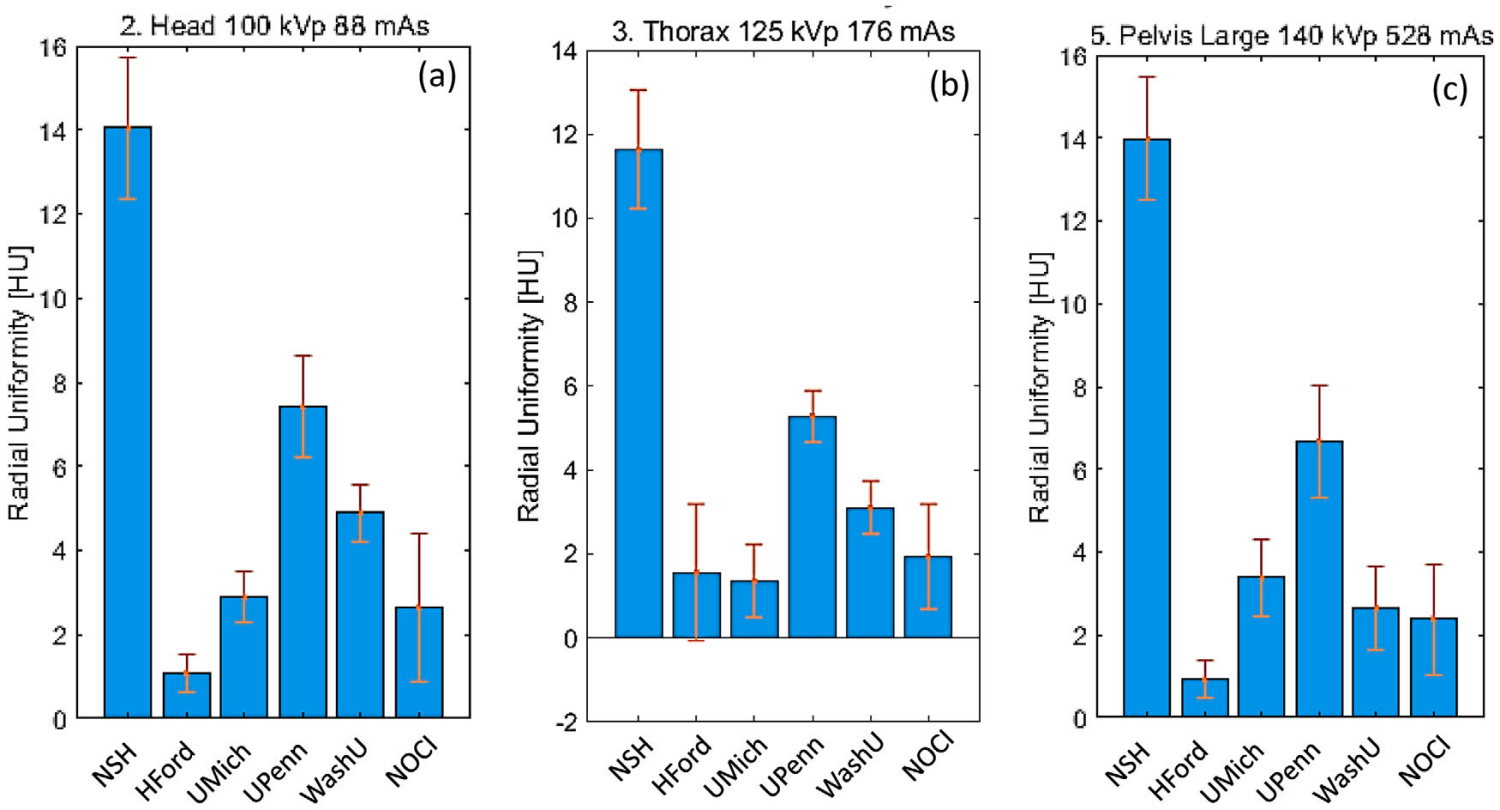
Uniformity comparison between periphery and center based on RU metrics using Catphan 604. RU, radial uniformity.

Uniformity is measured in terms of RU, UI for center‐periphery homogeneity, and InU for vertical and horizontal homogeneity. Figure 4 shows RU variation among the six institutions. Values ranged from 1 to 7 HU in the three protocols, although the value within each institution is self‐consistent; NSH and UPenn ranged from 5 to 7 HU, WashU and NOCI from 2 to 4 HU, and HFord and UMich from 1 to 3 HU. Although NSH shows larger results, a *z*‐score test gave values below 2 in the three protocols. The RU values are consistent with the literature values, which range from 5 to 10 HU.^3^, ^30^, ^31^ Similar reports with HyperSight systems showed larger values (10 to 18 HU), which increase with energy.^14^ The difference with our results can be given by the automatization software and the different reconstruction algorithms used. Our values are comparable to recently reported ACR phantom measurements,^13^ but slightly larger since the ACR phantom is smaller than the one used in this report.

Other uniformity parameters, such as UI, show more inter‐institutional consistency since they are normalized values. All uniformity metrics can be reviewed in the link provided in Appendix A.

**FIGURE 5:**
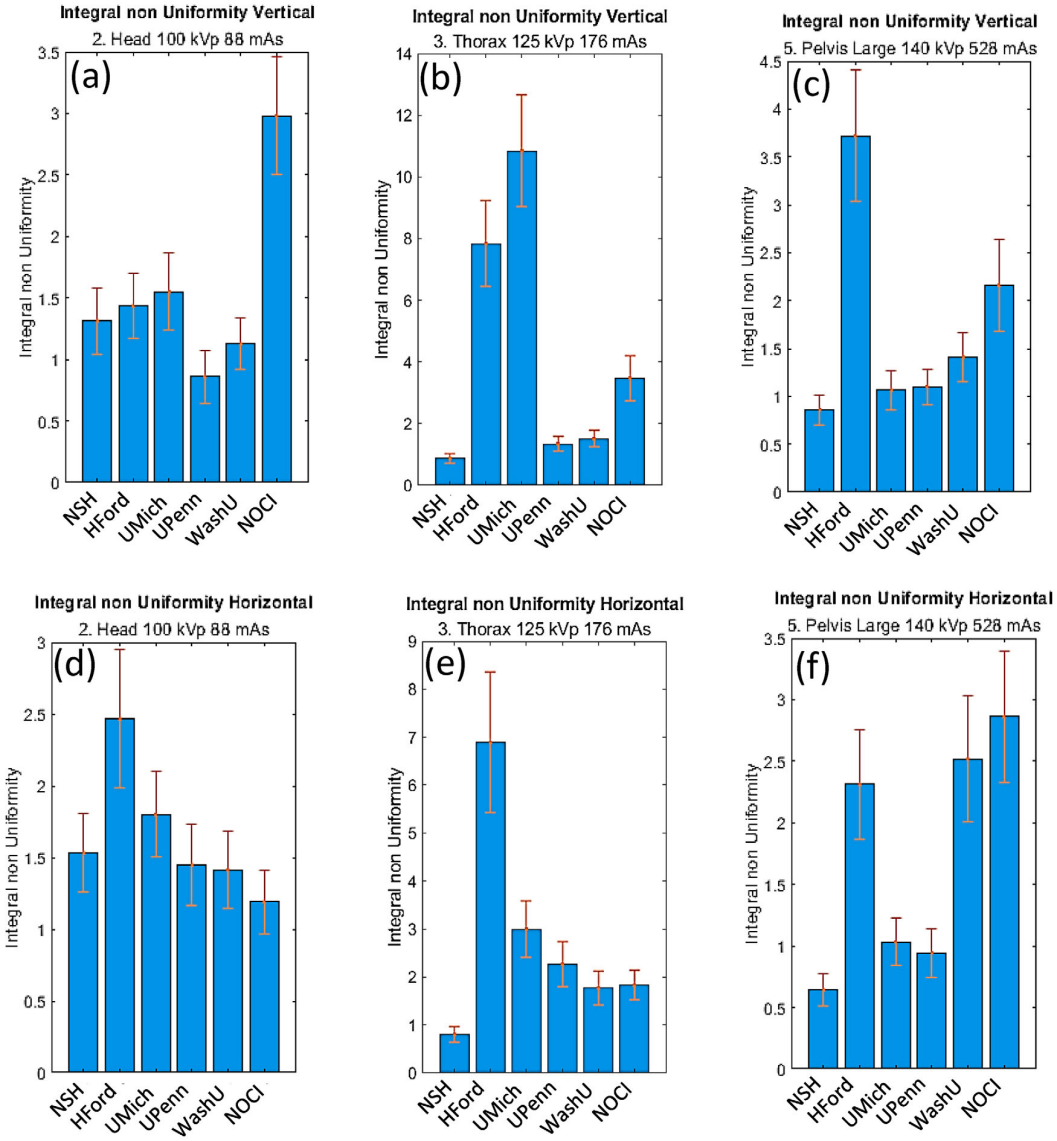
Integral nonuniformity results using Catphan 604. Vertical (top) and horizontal (bottom) integral nonuniformity.

The vertical and horizontal projection homogeneity was measured in terms of InU. At the lowest and highest energy, the values are consistent among most institutions, as shown in Figure 5a (mean 1.8 ± 0.6 HU) and Figure 5d (mean 1.7 ± 0.7 HU), and in Figure 5c (mean 1.8 ± 0.9 HU) and Figure 5f (mean 1.8 ± 1.0 HU), within the error values. However, at intermediate energies (125 kVp), as shown in Figure 5b (mean 2.8 ± 2.1 HU) and Figure 5e (mean 4.3 ± 4.1 HU), the InU varies significantly among institutions. No clear dependence on the energy level between the vertical and horizontal orientations has been observed.

#### 3.2.2 Low contrast

**FIGURE 6:**
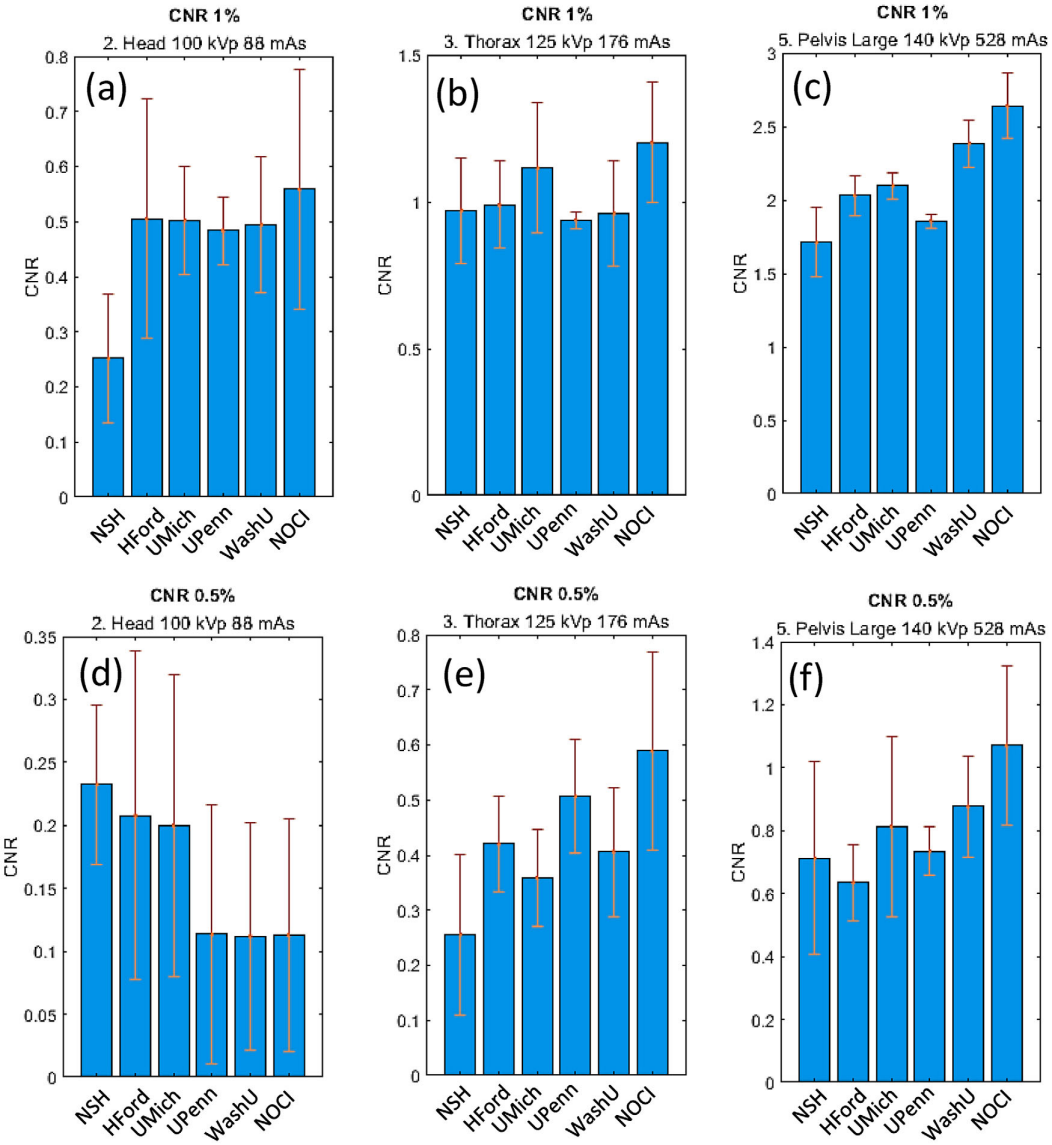
CNR for three protocols: head (a) and (d), thorax (b) and (e), and pelvis large (c) and (f). Two 15 mm diameter ROI were analyzed: the top row refers to the 1% contrast and bottom row refers to the 0.5 % contrast. CNR, contrast‐to‐noise ratio; ROI, region of interest.

The phantom consists of three sets of contrasts regions of 1%, 0.5%, and 0.3% relative to the background. While each set comprises different diameters, only the largest, 15 mm, was considered in this study, as smaller diameters often lacked sufficient signal‐to‐noise ratio for comparison across institutions and protocols. The link provided in Appendix A offers access to all results for comprehensive analysis. The CNR shown in Figure 6 shows 1% and 0.5% contrast for the three reference protocols. The 0.3 % contrast results are not presented in Figure 6 because it did not show consistent results due to its low signal‐to‐noise ratio, even with the 15 mm ROI. The top row shows the results for 1% contrast, with CNR values approximately double those presented in the bottom row, which shows the results for 0.5% contrast. As expected, relative error decreases as exposure increases. The precision also improves as energy and current increases. Values are larger than those reported with conventional CBCT with the same phantom and similar ROI.^32^, ^33^ Although a dependence with the square root of the milliampere‐seconds should be expected due to a better signal‐to‐noise ratio,^34^, ^35^ the CNR in both top and bottom rows can be approximated to a linear dependence within the 88 to 528 mAs range when using small contrast targets. Recent HyperSight reports showed similar CNR even using a different phantom (the ACR phantom has nominal contrast reference target of 0.6%).^13^

#### 3.2.3 Slice thickness

**FIGURE 7:**
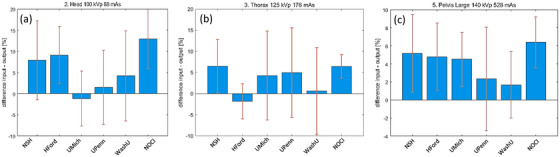
Slice thickness at the reference protocols: (a) head, (b) thorax and (c)pelvis large. These protocols were reconstructed with a 2 mm slice.

All protocols were reconstructed with a 2 mm slice thickness except for the CBCTp protocols, which were reconstructed with 3 mm slices. Figure 7 shows the percentage difference between the thickness of the reconstruction input slice thickness minus the measured FWHM, normalized to the input slice thickness. The measured thickness differences were within 5% in the case of 125 and 140 kVp protocols, see Figure 7b,c. The low energy protocol, in Figure 7a, shows larger differences due to a higher noise given by the low milliampere‐seconds. The other protocols showed a similar accuracy within the 5%; see more information in the link provided in Appendix A.

#### 3.2.4 Circular symmetry

Table 3 shows the measurements for the circular symmetry. Measurements showed good accuracy since the measured size and uncertainty is within the pixel size, which is the smallest error in all protocols; complete information in the link provided in Appendix A.

**TABLE 3:** Circular symmetry measurements of three protocols of the six institutions. Uncertainty is given by the standard deviation across five slices centered on section 3 of the Catphan phantom.

Institution	2. Head 100 kVp 88 mAs	3. Thorax 125 kVp 176 mAs	5. Pelvis large 140 kVp 528 mAs
NSH /(mm)	50.4 ± 0.4	50.4 ± 0.4	50.4 ± 0.4
HFord /(mm)	50.4 ± 0.4	50.4 ± 0.4	50.4 ± 0.4
UMich /(mm)	49.4 ± 0.1	49.4 ± 0.1	49.4 ± 0.1
UPenn /(mm)	49.7 ± 0.7	49.9 ± 0.7	49.7 ± 0.7
WashU /(mm)	50.4 ± 0.1	50.4 ± 0.1	50.4 ± 0.1
NOCI /(mm)	49.9 ± 0.7	49.9 ± 0.7	49.9 ± 0.7

Abbreviations: HFord, Henry Ford Cancer Institute; NOCI, Northeastern Oklahoma Cancer Institute; NSH, Nova Scotia Health Authority; UMich, The University of Michigan; UPenn, The University of Pennsylvania, WashU, Washington University.

#### 3.2.5 CT numbers

**FIGURE 8:**
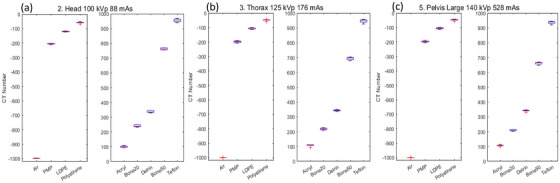
CT numbers for the reference protocols: (a) head, (b) thorax and (c) pelvis large. These protocols were reconstructed with a 2 mm slice. In the x‐axis the materials are shown. Note that the materials are ordered with increasing CT number, and it is not RED linearly depicted; moreover, there is not a linear dependence between RED and CT number in this phantom (see the manufacturer's manual for details^21^). CT, computed tomography; RED, relative electron density.

Monitoring sensitometry target values on CBCT over time can yield valuable insights into scanner performance variations. Figure 8 shows the CT numbers for various sensitometry targets in the CatPhan phantom across the three reference protocols. The values remained consistent within the ranges specified by the manufacturer and references in the literature.^14^, ^36^, ^37^ Like the AED phantom results, precision decreases as the RED of the sensitometry targets increases. Additionally, a slight reduction in CT numbers is observed as beam energy increases, a behavior that is consistently observed across all institutions. This behavior, as observed in the AED phantom, reduces the RED sensitivity of the HyperSight as energy increases. Detailed values can be found in the link provided in Appendix A.

#### 3.2.6 Spatial resolution

##### Modulation transfer function

**FIGURE 9:**
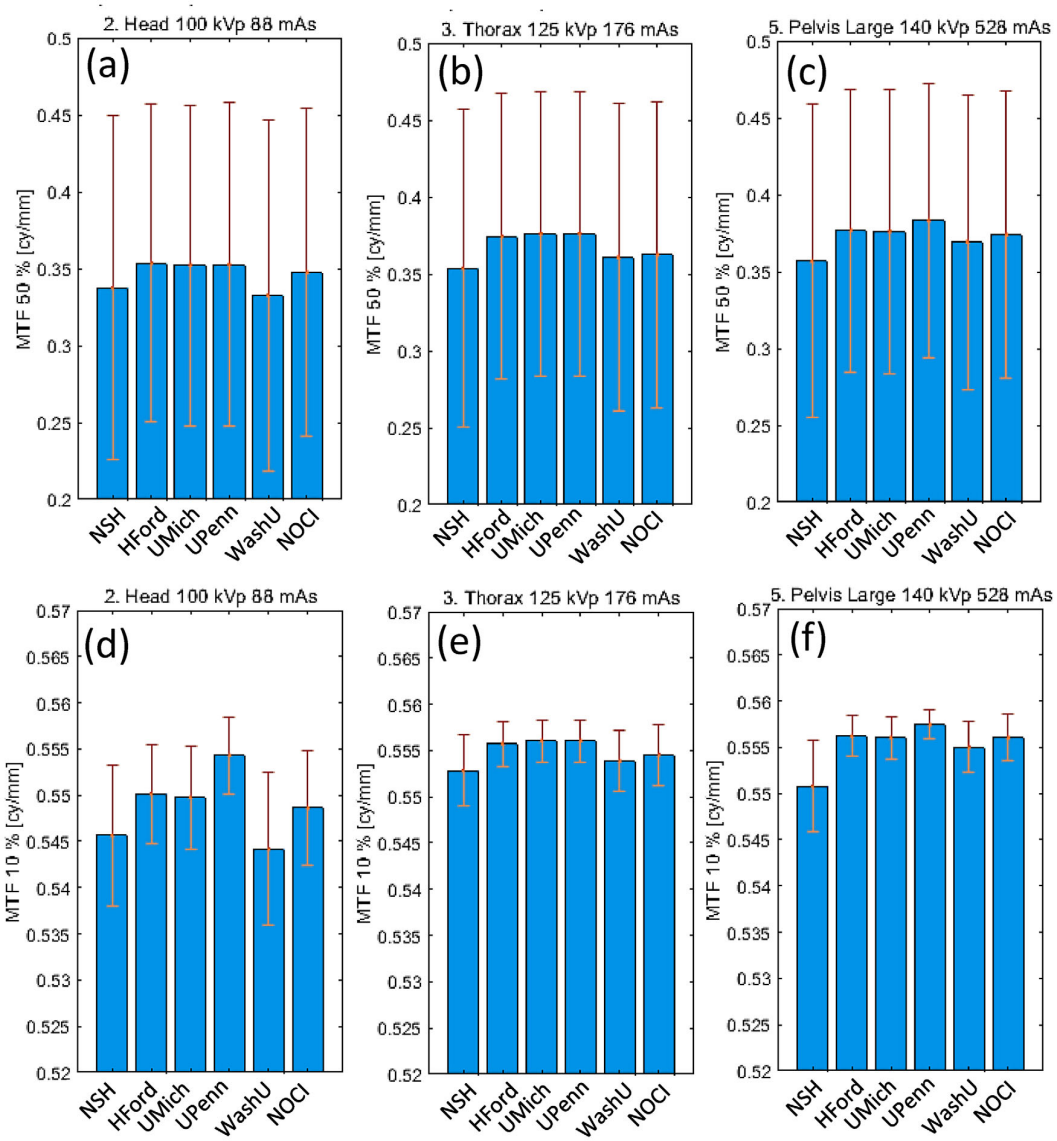
Spatial and contrast resolution in terms of MTF. The top row shows the value of the MTF at 50%, while the bottom row for MTF at 10% for the reference protocols. Since the MTF at 50% has a larger slope than at 10%, the error bars are larger. Note that the top and bottom rows have different vertical axis limits for the sake of clarity. MTF, modulation transfer function.

MTF was one of the most consistent measurements among protocols and institutions, primarily owing to its dependence on the pixel per mm setting during reconstruction and detector properties. MTF values at 10% and 50% were estimated by linear interpolation of the MTF function, see values in Figure 9. These values fall within the range of those reported in the literature.^31^, ^38^, ^39^ Although comparison can be difficult due to the lack of information regarding the analysis methodology. The closest comparable report^14^ showed similar values with an energy dependence that was not clearly observed in Figure 9. However, regardless of the analysis method used (line pair, point or line spread function), the results are comparable with literature.

##### Line pairs per cm

The spatial resolution metric evaluated was detectable line pairs per cm. A peak‐valley fitting algorithm was employed to ensure user‐independent comparison among institutions and protocols. The maximum line pairs per cm ranged from 2 to 5 lp/cm across institutions and protocols, as shown in Table 4. In protocols with low kilovoltage peak and milliampere‐seconds, such as the head protocol, one institution yielded 2 lp/cm while others showed 3 lp/cm. Conversely, in protocols with high kilovoltage peak and milliampere‐seconds, the higher signal‐to‐noise ratio enabled better peak differentiation, resulting in 3 and 4 lp/cm detection. It is noteworthy that UMich data has a remarkably good line pairs per cm ratio compared to the other institutions. This can be attributed to UMich's better alignment and slightly less noisy images. These factors influence the algorithm's ability to differentiate between peaks and valleys.

**TABLE 4:** Higher line pairs per cm detected by the fitting algorithm.

Institution	2. Head 100 kVp 88 mAs	3. Thorax 125 kVp 176 mAs	5. Pelvis large 140 kVp 528 mAs
NSH	3	3	3
HFrod	4	3	3
UMich	5	5	5
UPenn	4	4	4
WashU	2	3	3
NOCI	3	3	3

Abbreviations: HFord, Henry Ford Cancer Institute; NOCI, Northeastern Oklahoma Cancer Institute; NSH, Nova Scotia Health Authority; UMich, The University of Michigan; UPenn, The University of Pennsylvania; WashU, Washington University.

## 4 CONCLUSIONS

This study presents the findings of the first multi‐institutional analysis of HyperSight CBCT imaging performance employing the same phantoms across three Halcyon and three Ethos platforms located in six different institutions. We evaluated various key metrics, including spatial resolution, CNR, uniformity, CT numbers, and HU calibration curves. Results show consistency in RU, circular symmetry, integral nonuniformity, contrast, and spatial resolution. The findings are consistent with the available literature. Regarding the HU calibration curves, while institutions generally exhibit similar results at RED below 1, variations become noticeable at higher RED values, likely due to subtle differences in beam spectra across equipment. The *z*‐score was approximately 1.5 in all protocols at each RED value and has never exceeded 2 in absolute value. Additional research would be necessary to establish the causes and significance of this variability. The data herein should provide a benchmark for those commissioning the HyperSight system in assessing image quality and the HU‐to‐RED relationship. This study contributes valuable insights into the performance and reproducibility of the HyperSight CBCT imaging system in different clinical scenarios and highlights areas for further research and protocol optimization.

## AUTHOR CONTRIBUTIONS

Luis Agulles‐Pedrós: Protocol analysis, image acquisitions and analysis, and manuscript development. R Lee MacDonald: Protocol design and review, and image acquisitions, and manuscript review. Amanda Jean Cherpak: Protocol design and review, and image acquisitions, and manuscript review. Nayha Dixit: Protocol design and review, and image acquisitions, and manuscript review. Lei Dong: Protocol design and review, and image acquisitions, and manuscript review. Tianyu Zhao: Protocol design and review, and image acquisitions, and manuscript review. Kundan Thind: Protocol design and review, and image acquisitions, and manuscript review. Anthony Doemer: Protocol design and review, and image acquisitions, and manuscript review. Boon‐Keng Teo: Protocol design and review, and image acquisitions, and manuscript review. Shiqin Su: Protocol design and review, and image acquisitions, and manuscript review. Alexander Moncion: Protocol design and review, and image acquisitions, and manuscript review. James L. Robar: PI of the project: Protocol design and review, and image acquisitions, and manuscript review.

## CONFLICT OF INTEREST STATEMENT

James L. Robar, Amanda Cherpak, and Robert Lee MacDonald report honoraria, consulting fees, and support for attending meetings and holding research grants with financial support from Varian Medical Systems. James L. Robar also cochairs the Varian Intelligent Imaging Consortium, which focuses on HyperSight technology. Nayha Dixit works at Varian.

## Supporting information



Supporting Information

## APPENDIX A

### A.1

See link for complete results consultation: https://data.mendeley.com/datasets/27456wjx5s/4.
