# Supplementary material for: Multi‐institutional study on image quality for a novel CBCT solution on O‐ring linac
Source: J Appl Clin Med Phys. 2025 Mar 6;26(6):e70023. doi: 10.1002/acm2.70023 (PMC12148799; doi:10.1002/acm2.70023)
Supplement: Supplementary file 1 — Supporting Information [file ACM2-26-e70023-s001.docx]

Appendix instructions:

**Manual for Accessing and Interpreting the Data in Tables.zip**

This document outlines the steps to access, organize, and understand the data in the Tables.zip file. This manual is designed to support the accompanying paper and ensure proper dataset understanding.

**1. Downloading and Locating the Dataset**

1. **Access the Data:** The dataset is hosted on Mendeley Data. Use the following link to download the Tables.zip file: <https://data.mendeley.com/datasets/56jz42bkst/2>.
2. **Extract the File:** Once downloaded, extract Tables.zip into a local directory. Within the extracted folder, locate a subfolder named REDvsHU. This folder contains the primary Excel tables for analysis.

**2. Structure of the Tables in REDvsHU Folder**

The folder contains Excel files named using the format:

Tab_XXX_YYYY.xlsx

- **XXX:** Describes the type of data within the table. This can have four designations:
  - **AED0:** Data for ROIs with Relative Electron Density (RED) values ≤ 1.
  - **AED1:** Data for ROIs with RED values > 1.
  - **RED_Hi:** Results from linear fitting of RED vs HU for RED > 1.
  - **RED_Lo:** Results from linear fitting of RED vs HU for RED ≤ 1.
- **YYYY:** Specifies the imaging protocol, including kV, mAs, and slice thickness. These protocols are as follows:

| **Protocol** | **kV** | **mAs** | **Slice Thickness** |
| --- | --- | --- | --- |
| 1. Breast | 125 | 29 | 2 mm |
| 2. Head | 100 | 88 | 2 mm |
| 3. Thorax | 125 | 176 | 2 mm |
| 4. Pelvis | 125 | 469 | 2 mm |
| 5. Pelvis Large | 140 | 528 | 2 mm |
| 6. CBCTp Head* | 125 | 804 | 3 mm |
| 7. CBCTp Pelvis* | 125 | 528 | 3 mm |
| 8. CBCTp Abdomen Lg* | 140 | 880 | 3 mm |

Protocols marked with an asterisk (*) are CBCTp protocols designed for planning purposes.

**3. Understanding the AED Tables**

The tables with **AED0** and **AED1** nomenclature contain raw data from the Advanced Electron Density phantom. These tables include:

- **Columns:**
  - **RED (value in parentheses):** The mean Relative Electron Density for the ROI.
  - **Standard Deviation (SD):** Across five axial-centered slices.
- **Rows:** Each row corresponds to data from a specific institution.

**4. Understanding the RED_Hi and RED_Lo Tables**

The **RED_Hi** and **RED_Lo** tables provide results from the two linear fitting of RED vs HU:

- **RED_Hi:** Fitting for RED > 1.
- **RED_Lo:** Fitting for RED ≤ 1.

The fitting follows the equation:

$$RED=\left( HU\times slope \right)+intercept$$

These tables provide slope and intercept values for each institution.

**5. Additional Image QC Data**

Beyond REDvsHU, other subfolders, such as Prot1Breast, Prot2Head, etc., contain image quality control (QC) data. These datasets were analyzed following the procedures detailed in the accompanying paper.

**6. References and Further Information**

For more details on data processing and interpretation, refer to the draft of the paper included in the dataset. If additional clarification is needed, feel free to reach out to the authors.
